# Whole genome sequence data of a lignocellulose-degrading bacterium, Arthrobacter koreensis BSB isolated from the soils of Santiniketan, India

**DOI:** 10.1016/j.dib.2024.110915

**Published:** 2024-09-12

**Authors:** Binoy Kumar Show, Andrew B. Ross, Raju Biswas, Shibani Chaudhury, Srinivasan Balachandran

**Affiliations:** a Department of Environmental Studies, Siksha-Bhavana, Visva-Bharati, Santiniketan 731235, West Bengal, India; b School of Chemical and Process Engineering, University of Leeds, Leeds, LS2 9JT, United Kingdom; c Department of Botany, Siksha-Bhavana, Visva-Bharati, Santiniketan 731235, West Bengal, India

**Keywords:** Cellulase, Enzyme, Lignocellulose, Arthrobacter koreensis BSB

## Abstract

A draft genome sequence of an isolate of *Arthrobacter koreensis* BSB from Santiniketan soil is being published. A. koreensis BSB produces lignocellulases, which are crucial in plant biomass degradation. It is a potential source of enzymes of digestive importance, especially lignocellulases. Genomic DNA was isolated from a single bacterial colony using a QIAgen Blood and Tissue kit (QIAgen Inc., Canada). Illumina HiSeq X performed the DNA sequence, employing 2 × 150 paired-end chemistry, and 8,725,587 reads were obtained, corresponding to a sequence coverage of 755X. The draft genome assembly formed 15 contigs > 200 base pairs in length (N50 value= 446, 958 and L50= 3). The genome size is 3,466,004 base pairs with an average GC percentage of 65.94 %. Annotation and prediction of genes were carried out with Prokka v.1.14.6, and 3,172 CDS, 3236 genes, 58 tRNA genes, 4 rRNA genes, and 2 tmRNA genes were identified.

Specifications Table

**Table:** 

Subject	Microbiology • Applied Microbiology
Specific subject area	Omics: Genomics
Type of data	Table, Figure Raw, Analyzed, Filtered, Deposited
Data collection	Genomic DNA was isolated from a single colony using the QIAgen Blood and Tissue Kit (QIAgen Inc., Canada). Sequencing was performed using the Illumina HiSeq X platform. After sequencing, quality control was performed with FastQC, trimming, and size selection with Cutadapt v2.9. The genome was de novo assembled using Unicycler v0.4.4, with assembly summary statistics performed by QUAST v5.1.0. The assembly pipeline includes BayesHammer, and the assembly itself was completed using SPAdes v3.13.0. Genome annotation and gene prediction were performed using Procca v1.14.6. The PANZER webserver was used to annotate the proteins functionally.
Data source location	The BSB strain was extracted from the soils of Santiniketan in West Bengal, India, located at 23.40’22” N latitude and 87.39’44” E longitude.
Data accessibility	Repository name: NCBI (National Center for Biotechnology Information) GenBank Nucleotide database Data identification number: BioProject accession number PRJNA890884 Direct URL to data: https://www.ncbi.nlm.nih.gov/bioproject/PRJNA890884 https://www.ncbi.nlm.nih.gov/sra/SRR22254257

## 1 Value of the Data

• Whole genome sequence of the *A. koreensis* BSB could be valuable for researching the ecology and taxonomy of bacteria, especially in taxonomic identification and dispersion.
• The data in this article may be of interest to researchers working in the domains of environmental microbiology, environmental biotechnology, genomics, and renewable energy.
• The *A. koreensis* strain BSB genome sequence data may prove valuable to researchers who wish to do comparative genomic analysis between various strains and environments.

## 2 Background

The bacterium *Arthrobacter koreensis* belongs to the family *Micrococcaceae*. This genus is a Gram-positive, rod-shaped bacteria that is well known for its abundance in soil and capacity to withstand extreme circumstances [1]. Organic waste biomass and other noxious weeds were not effectively utilized during anaerobic digestion (AD) due to their lignocellulosic complexity, structural recalcitrance, and hydrophobic characteristics, which include numerous biologically stable linkages [2]. Lignocellulosic biomass comprises cellulose, hemicellulose, and lignin, among other polymeric components. The primary constituent of lignocellulose is cellulose, which is protected in a hemicellulose matrix, with lignin comprising the outermost layer [3]. Studies on microbial degradation have shown a variety of microorganisms with potent enzymes capable of breaking down lignocellulose [4]. The direct breakdown of polymeric lignocellulose by soil microorganisms via a variety of active enzymes that are active in a broad range of pH and temperature conditions emphasizes the importance of researching these consortia in biological contexts. The present study is based on sequencing and analysis of the draft genome of *A. koreensis* BSB.

## 3 Data Description

**Fig. 1:**
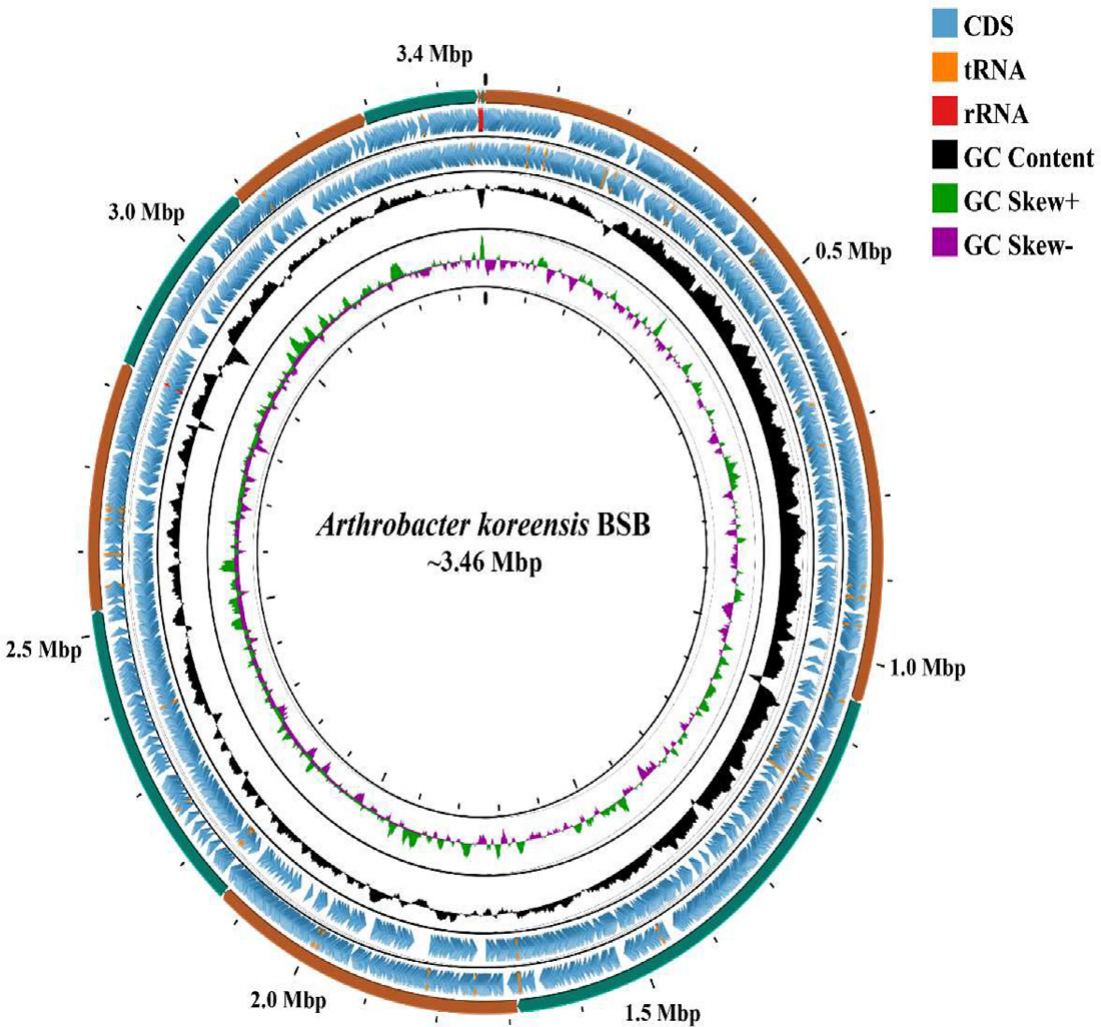
Graphical presentation of A. koreensis BSB genome (∼3.46 Mbp) using CG view server. The concentric circle from outer to inner represents the following: circle 1: DNA base position (Mbp); circles 2 contigs; circles 3 and 4, protein-coding genes on forward and reverse strands; circle 5 (black): G + C content; circle 6: G + C skew.

The whole genome sequence of *Arthrobacter koreensis* strain BSB, which contains lignocellulases genes involved in plant biomass degradation, is given in this article. The assembled genome sequence of *A. koreensis* strain BSB comprises 15 contigs larger than 200 base pairs (bp), with N50 of 446,958 bp and L50 of 3. The assembled genome is nearly complete with low contamination (100 % completeness, 0.00 % contamination), and the sequencing coverage is 755X. The total size of the genome is 3466,004 bp with an average GC content of 65.94 %. A total of 3172 coding sequences (CDS), 3236 (genes), 58 tRNA genes, 4 rRNA genes, and 2 tmRNA genes were identified in the genome annotation (Table 1) (Fig. 1).

**Table 1:** General genomic features of *Arthrobacter* sp. BSB.

Feature	Value
Genome size (bp)	3466,004
G + C content (%)	65.94
No. of contigs	15
N50	446,958
N90	204,120
L50	3
L90	7
CDS (coding sequences)	3172
tRNA	58
rRNA	4
tmRNA	2

**Fig. 2:**
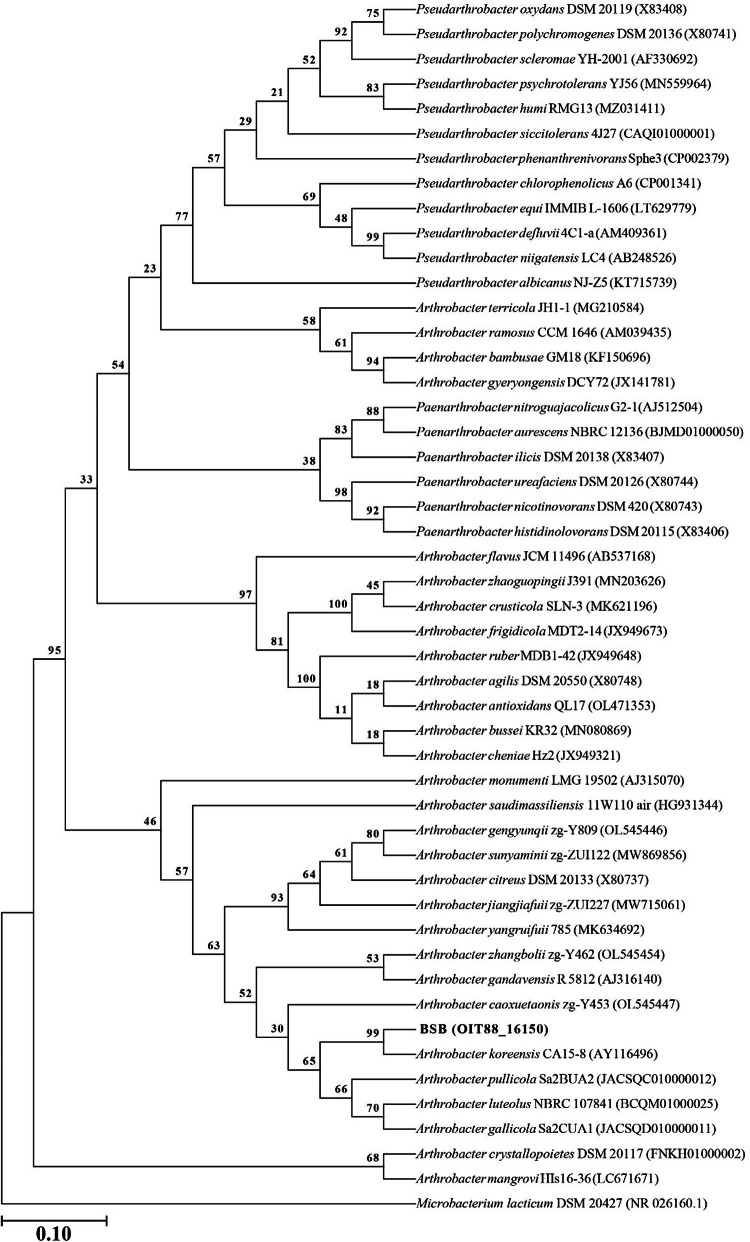
Phylogenetic tree generated from the 16S rRNA gene sequences of Arthrobacter koreensis BSB and the closely related species of Arthrobacter sp. Bootstrap values (>50 % are expressed as percentages of 1000 replications) are shown at branching points (A scale of 0.10 suggests the sequences being compared have a 10 % nucleotide difference on average).

**Fig. 3:**
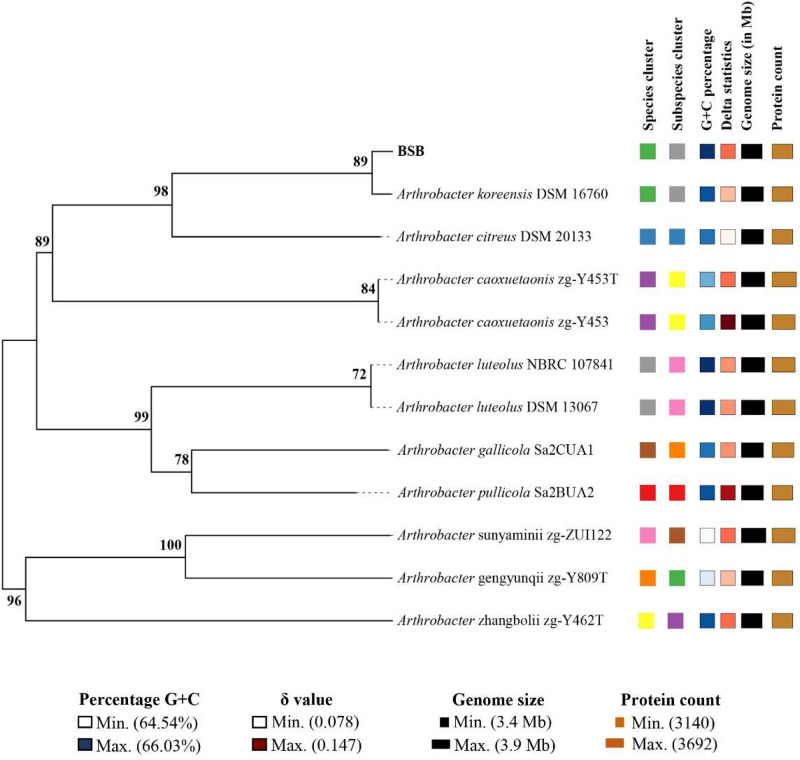
TYGS tree based on whole genome sequence inferred with FastME 2.1.6.1 from Genome Blast Distance Phylogeny (GBDP) distances calculated from genome sequences. The branch lengths are scaled in terms of the GBDP distance formula d5. The numbers above branches are GBDP pseudo-bootstrap support values > 60 % from 100 replications, with an average branch support of 89.4 %. The tree was rooted at the midpoint.

The 16S rRNA gene sequence of strain BSB has more than 97 % similarity with several members of the family *Micrococcaceae*, including 22 species of the genera *Arthrobacter* and *Paenarthrobacter* (Supplementary Table S1). Among them, the phylogenetic nearest species is *A. koreensis*, with 99.51 % sequence similarity. The maximum-likelihood tree of the 16S rRNA gene sequence showed that strain BSB clustered in the same clade as *A. koreensis* (Fig. 2). Moreover, the phylogenetic tree based on the whole genome sequence revealed that strain BSB has a high bootstrap value (89 %) with *A. koreensis* (Fig. 3). Digital whole genome comparisons are widely regarded as the definitive method for determining the systematic classification of a species [5]. The digital DNA-DNA hybridization (dDDH) value of strain BSB is 93.5 %, which further validated the strains BSB and *A. koreensis* are closely related.

The functional annotation analysis of the PANZER webserver suggested that strain BSB harbors multiple genes involved in cellulase production. The strain BSB genome contains 20 lignocellulose-degrading enzyme genes (Supplementary Table S2 and Supplementary Table S3).

## 4 Experimental Design, Materials and Methods

### 4.1 Isolation of *arthrobacter koreensis* strain BSB

*Arthrobacter koreensis* strain BSB was isolated from soil samples collected in Santiniketan, West Bengal, India (coordinates: 23.40’22” N, 87.39’44” E). The strain was obtained through the standard serial dilution method and cultured on Bushnell Haas agar medium with 1 % (w/v) carboxymethylcellulose (BH—CMC) and incubated for 72 hrs at 37 °C. Subsequent culturing was performed in a minimal salt medium (MSM) [NaNO_3_, 2.50 (g/l); KH_2_PO_4_, 2.0 (g/l); MgSO_4_, 0.20 (g/l); NaCl, 0.20 (g/l); CaCl_2_, 0.10 (g/l); Agar-agar, 20.0 (g/l); medium pH 7.0 ± 0.2] containing 1 % (w/v) alkaline lignin (TCI, Japan). The strain underwent repeated streaking on BH—CMC agar plates for further purification and was incubated for 48 h at 37 °C [6].

### 4.2 Genomic DNA extraction

According to the manufacturer's protocol, genomic DNA was isolated from the bacterial culture utilizing the Qiagen Blood and Tissue kit (QIAgen Inc., Canada). The quality, determined by the OD260/280 ratio, and the concentration of the extracted DNA were measured using an Agilent BioTek Epoch 2 microplate reader (USA) [7].

### 4.3 Whole genome sequencing and assembly

The genome of strain BSB was sequenced using the Illumina HiSeq X platform with 2 × 150 bp paired-end reads, generating 8725,587 raw reads. Initial quality checks of the sequences were performed with FastQC v0.11.9 [8]. Reads were then trimmed and filtered to retain those longer than 200 bp using Cutadapt v2.9 [9], resulting in 8722,080 high-quality paired reads. Genome assembly was conducted using Unicycler v0.4.4 with default parameters, incorporating BayesHammer [10] for error correction and SPAdes v3.13.0 [11] for assembly, with the k-mer set to 99. The draft genome's quality was evaluated using CheckM v1.1.6 and QUAST v5.1.0. A circular representation of the draft genome was generated using the CGView server (https://www.cgview.ca) [12] (Fig. 1). Annotation and gene prediction were performed with Prokka v1.14.6. After completing the annotation with Prokka, the protein sequences were further annotated using the PANNZER2 web server [13].

### 4.4 Phylogenetic analysis

The near-complete 16S rRNA gene sequence was extracted from the whole genome and compared with type strains via the EzBioCloud server (www.ezbiocloud.net) [14]. Sequences from closely related type taxa were retrieved from the NCBI database (www.ncbi.nlm.nih.gov) and aligned using the MUSCLE algorithm in MEGA X [15]. The complete deletion option was utilized to address gaps and missing data, while the Tamura 3-parameter model with Gamma distribution and invariable sites (T92+*G* + *I*) was applied for nucleotide substitution modeling [16]. Maximum-likelihood phylogenetic trees were then constructed with 1000 bootstrap replications in MEGA X (see Fig. 2). For whole genome-based taxonomic analysis, including genome-to-genome distances (GGDs) and digital DNA-DNA hybridization (dDDH), the Type Strain Genome Server (TYGS) (https://www.tygs.dsmz.de) [17] was employed (see Fig. 3). The whole-genome phylogenetic tree was created using FastME [18], based on genome BLAST distance phylogeny (GBDP), and was rooted at the midpoint [19].

## Limitations

Not applicable.

## Ethics Statement

The authors have read and follow the ethical requirements for publication in Data in Brief and confirming that the current work does not involve human subjects, animal experiments, or any data collected from social media platforms.

## Credit Author Statement

**Binoy Kumar Show:** Conceptualization, Data curation, Formal analysis, Methodology, Validation, Writing – original draft | **Andrew B. Ross:** Conceptualization, Investigation, Project administration, Supervision, Writing – review and editing | **Raju Biswas:** Data curation, Formal analysis, Resources, Writing – original draft | **Shibani Chaudhury:** Conceptualization, Investigation, Project administration, Supervision, Writing – review and editing | **Srinivasan Balachandran:** Conceptualization, Investigation, Project administration, Supervision, Writing – review and editing.

## Data Availability

Isolation of lignocellulose degrading bacteria from soil of Shantiniketan, West Bengal, India (Original data) (NCBI).

## Appendix Supplementary materials

Image, application 1

Image, application 2

Image, application 3
