# Supplementary material for: Whole genome sequence data of a lignocellulose-degrading bacterium, Arthrobacter koreensis BSB isolated from the soils of Santiniketan, India
Source: Data Brief. 2024 Sep 12;57:110915. doi: 10.1016/j.dib.2024.110915 (PMC11424791; doi:10.1016/j.dib.2024.110915)
Supplement: Supplementary file 1 [file mmc1.docx]

**Supplementary materials to**

***Whole genome sequence data of a lignocellulose-degrading bacterium, Arthrobacter koreensis* BSB *isolated from the soils of Santiniketan, India***

***Binoy Kumar Show^1^, Andrew B. Ross^2^, Raju Biswas^3^, Shibani Chaudhury^1^, Srinivasan Balachandran^1^****

^1^ Department of Environmental Studies, Siksha-Bhavana, Visva-Bharati, Santiniketan 731235, West Bengal, India ([binoyshow@gmail.com](mailto:binoyshow@gmail.com), ORCID ID: 0000-0002-6870-7155; [shibani.chaudhury@visva-bharati.ac.in](mailto:shibani.chaudhury@visva-bharati.ac.in), ORCID ID: [0000-0001-7413-](https://orcid.org/0000-0002-2102-8809)6320; [s.balachandran@visva-bharati.ac.in](mailto:s.balachandran@visva-bharati.ac.in), ORCID ID: 0000-0003-4247-408X)

^2^School of Chemical and Process Engineering, University of Leeds, Leeds, LS2 9JT, United Kingdom

([A.B.Ross@leeds.ac.uk](mailto:A.B.Ross@leeds.ac.uk))

^3^ Department of Botany, Siksha-Bhavana, Visva-Bharati, Santiniketan 731235, West Bengal, India ([rajubiswas.26041995@gmail.com](mailto:rajubiswas.26041995@gmail.com), ORCID ID: 0000-0002-6131-7015)

****Corresponding author:*** [***s.balachandran@visva-bharati.ac.in***](mailto:s.balachandran@visva-bharati.ac.in)*.*

| Table S1. Ezbiocloud based 16S rRNA gene sequence similarity of strain MR1 with available type strain sequences in the database. | | | | | | |
| --- | --- | --- | --- | --- | --- | --- |
| Sl. No. | Taxon name | Strains names | Pairwise Similarity (%) | Mismatch/Total nt | Completeness (%) | Accession |
| 1 | *Arthrobacter luteolus* | NBRC 107841 | 99.59 | 6/1450 | 100 | BCQM01000025 |
| 2 | *Arthrobacter pullicola* | Sa2BUA2 | 99.59 | 6/1450 | 100 | JACSQC010000012 |
| 3 | *Arthrobacter gallicola* | Sa2CUA1 | 99.52 | 7/1450 | 100 | JACSQD010000011 |
| 4 | *Arthrobacter koreensis* | CA15-8 | 99.52 | 7/1449 | 100 | AY116496 |
| 5 | *Arthrobacter zhangbolii* | zg-Y462 | 99.17 | 12/1450 | 100 | OL545454 |
| 6 | *Arthrobacter caoxuetaonis* | zg-Y453 | 99.17 | 12/1450 | 100 | OL545447 |
| 7 | *Arthrobacter gandavensis* | R 5812 | 99.03 | 14/1442 | 99.52 | AJ316140 |
| 8 | *Arthrobacter gengyunqii* | zg-Y809 | 98.69 | 19/1449 | 100 | OL545446 |
| 9 | *Arthrobacter sunyaminii* | zg-ZUI122 | 98.62 | 20/1449 | 100 | MW869856 |
| 10 | *Arthrobacter citreus* | DSM 20133 | 98.55 | 21/1448 | 100 | X80737 |
| 11 | *Arthrobacter jiangjiafuii* | zg-ZUI227 | 98.14 | 27/1449 | 100 | MW715061 |
| 12 | *Arthrobacter yangruifuii* | 785 | 98.07 | 28/1449 | 100 | MK634692 |
| 13 | *Arthrobacter saudimassiliensis* | 11W110_air | 97.79 | 32/1446 | 100 | HG931344 |
| 14 | *Paenarthrobacter nitroguajacolicus* | G2-1 | 97.78 | 32/1443 | 100 | AJ512504 |
| 15 | *Paenarthrobacter aurescens* | NBRC 12136 | 97.71 | 33/1443 | 100 | BJMD01000050 |
| 16 | *Paenarthrobacter ilicis* | DSM 20138 | 97.36 | 38/1442 | 100 | X83407 |
| 17 | *Arthrobacter ruber* | MDB1-42 | 97.26 | 38/1388 | 96.09 | JX949648 |
| 18 | *Arthrobacter frigidicola* | MDT2-14 | 97.24 | 40/1447 | 100 | JX949673 |
| 19 | *Paenarthrobacter nicotinovorans* | DSM 420 | 97.22 | 40/1440 | 100 | X80743 |
| 20 | *Arthrobacter zhaoguopingii* | J391 | 97.10 | 42/1447 | 100 | MN203626 |
| 21 | *Arthrobacter crusticola* | SLN-3 | 97.09 | 42/1445 | 100 | MK621196 |
| 22 | *Arthrobacter monumenti* | LMG 19502 | 97.03 | 43/1447 | 100 | AJ315070 |
| 23 | *Arthrobacter ramosus* | CCM 1646 | 96.96 | 41/1350 | 93.49 | AM039435 |
| 24 | *Arthrobacter agilis* | DSM 20550 | 96.89 | 45/1445 | 100 | X80748 |
| 25 | *Arthrobacter bussei* | KR32 | 96.89 | 45/1445 | 100 | MN080869 |
| 26 | *Arthrobacter cheniae* | Hz2 | 96.89 | 45/1445 | 100 | JX949321 |
| 27 | *Paenarthrobacter histidinolovorans* | DSM 20115 | 96.87 | 45/1439 | 100 | X83406 |
| 28 | *Pseudarthrobacter psychrotolerans* | YJ56 | 96.80 | 44/1376 | 95.17 | MN559964 |
| 29 | *Arthrobacter bambusae* | GM18 | 96.78 | 45/1397 | 96.75 | KF150696 |
| 30 | *Arthrobacter gyeryongensis* | DCY72 | 96.77 | 45/1392 | 96.27 | JX141781 |
| 31 | *KI519454_s* | 35W | 96.75 | 47/1447 | 100 | KI519454 |
| 32 | *Arthrobacter antioxidans* | QL17 | 96.71 | 44/1338 | 92.79 | OL471353 |
| 33 | *Arthrobacter crystallopoietes* | DSM 20117 | 96.61 | 49/1446 | 100 | FNKH01000002 |
| 34 | *ANPE_s* | BAB-32 | 96.61 | 49/1445 | 100 | ANPE02000028 |
| 35 | *Arthrobacter mangrovi* | HIs16-36 | 96.53 | 50/1442 | 100 | LC671671 |
| 36 | *VSLD_s* | P9 | 96.48 | 51/1447 | 100 | VSLD01000019 |
| 37 | *Pseudarthrobacter humi* | RMG13 | 96.47 | 51/1444 | 100 | MZ031411 |
| 38 | *Pseudarthrobacter oxydans* | DSM 20119 | 96.40 | 52/1444 | 100 | X83408 |
| 39 | *Pseudarthrobacter chlorophenolicus* | A6 | 96.39 | 52/1442 | 100 | CP001341 |
| 40 | *Pseudarthrobacter phenanthrenivorans* | Sphe3 | 96.33 | 53/1444 | 100 | CP002379 |
| 41 | *Pseudarthrobacter equi* | IMMIB L-1606 | 96.33 | 53/1443 | 100 | LT629779 |
| 42 | *Paenarthrobacter ureafaciens* | DSM 20126 | 96.32 | 53/1441 | 100 | X80744 |
| 43 | *Pseudarthrobacter defluvii* | 4C1-a | 96.32 | 53/1441 | 99.79 | AM409361 |
| 44 | *Pseudarthrobacter scleromae* | YH-2001 | 96.32 | 52/1413 | 98.27 | AF330692 |
| 45 | *Pseudarthrobacter albicanus* | NJ-Z5 | 96.29 | 52/1401 | 97.03 | KT715739 |
| 46 | *Pseudarthrobacter niigatensis* | LC4 | 96.26 | 54/1444 | 100 | AB248526 |
| 47 | *Arthrobacter terricola* | JH1-1 | 96.26 | 54/1444 | 100 | MG210584 |
| 48 | *Pseudarthrobacter siccitolerans* | 4J27 | 96.20 | 55/1446 | 100 | CAQI01000001 |
| 49 | *Arthrobacter flavus* | JCM 11496 | 96.19 | 55/1445 | 100 | AB537168 |
| 50 | *Pseudarthrobacter polychromogenes* | DSM 20136 | 96.19 | 55/1445 | 100 | X80741 |
